# Bilateral Eagle’s Syndrome: A Case Report

**DOI:** 10.7759/cureus.77718

**Published:** 2025-01-20

**Authors:** Laliytha K Bijai, Fahad K Almasoudi, Firas Mansour M Almoallem, Rawaf F Alghamdi, Eyad A Assiri, Abdullah S Almedlej, Rahaf Y Mahanshi

**Affiliations:** 1 Maxillofacial Surgery and Diagnotic Sciences, King Saud bin Abdulaziz University for Health Sciences, Riyadh, SAU; 2 Ministry of National Guard Health Affairs, King Abdullah International Medical Research Center, Riyadh, SAU; 3 College of Dentistry, King Saud bin Abdulaziz University for Health Sciences, Riyadh, SAU

**Keywords:** cervicofacial pain, eagle's syndrome, stylocarotid artery syndrome, styloid process, sylohyoid ligament

## Abstract

Diagnosing a headache can be challenging for a physician and even more difficult for a dentist. Eagle’s syndrome, caused by an abnormal elongation of the styloid process, may result in headaches. Radiographic imaging is crucial for diagnosis. A 24-year-old male came in with pain on the left side of his forehead and neck. Despite numerous consultations with various doctors, a definitive diagnosis was never made. We conducted a thorough examination, followed by an orthopantomogram (OPG) and cone-beam computed tomography (CBCT), which revealed a bilateral elongated styloid process. Consequently, we diagnosed the patient with bilateral Eagle's syndrome based on his history, clinical examination, and radiographic findings. The diagnosis of Eagle’s syndrome is often overlooked due to misleading and overlapping symptoms, leading patients to consult multiple doctors without finding relief from their condition. A case of bilateral Eagle’s syndrome is presented through a comprehensive examination and essential radiographic imaging techniques. This report highlights the importance of accurate and timely diagnosis to improve a patient’s quality of life.

## Introduction

Eagle's syndrome is a rare condition, first described by Watt W. Eagle, that is caused by an elongated or ossified styloid process [1,2]. The styloid process is a slender bone arising from the petrous part of the temporal bone. The incidence of this condition is estimated at 4% of the population, of which less than 0.2% of patients are symptomatic [3]. There are two types of Eagle’s syndrome: classic Eagle’s and stylocarotid artery syndrome [2]. The former is often observed post-tonsillectomy. The latter occurs due to impingement on nerve plexuses, leading to neurological symptoms such as vision disturbances or transient ischemic attacks. An elongated styloid process may result in recurrent throat and neck pain that can radiate to the ear [1]. Patients may also experience dysphagia, headaches, or vascular symptoms like dizziness or syncope from compression of the carotid artery [1,4].

The diagnosis typically involves radiographic imaging to evaluate the length and angulation of the styloid process, often supported by clinical palpation of the tonsillar fossa for confirmation [4]. Following tonsillectomy, the patient may experience a foreign body sensation in the throat as the styloid process lies immediately posterior to the tonsillar fossa. Moreover, upon visual examination, the patient is expected to look normal. Thus, the history, findings elicited on palpation, and radiographic evidence will aid in the diagnosis. Differential diagnoses for this condition may include pharyngitis, tonsillitis, or temporomandibular joint disorders [1,4]. Management typically includes conservative approaches, such as analgesics or corticosteroid injections. Unfortunately, some patients do not find relief with conservative management and may require surgical interventions, such as a styloidectomy via transoral or transcervical approaches [5]. A 24-year-old male reported bilateral Eagle’s syndrome and presented with headaches and discomfort localized to the neck. Due to the masking and inconsistent symptoms, diagnosing this condition was difficult. Patients often consult multiple specialists without relief from pain, making it even more frustrating when the pain does not respond to analgesics. This report emphasizes the significance of accurate diagnosis and suitable intervention in improving patient outcomes.

## Case presentation

Case history

A 24-year-old male presented to the dental clinic with pain localized to the left side of the forehead and the neck area. The onset of pain was three years ago when he had sharp headaches localized to the left side of the forehead. These headache episodes lasted an entire day, with no relieving factors other than sleep. For the last few months, the pain was aggravated when sleeping on the back of his head with relief on anterior flexion of the neck. Turning the head to the left elicits significant pain; however, turning to the right provides partial relief from the right side, but the pain on the left remains mildly discomforting. The patient also mentioned that he cannot sleep on his back; instead, he sleeps face down with his left cheek against the pillow to alleviate the discomfort. Occasionally, the patient experiences electric shock-like sensations in the back of the neck. These symptoms occurred more than twice a week. The patient had visited a hospital three years back for this complaint and was diagnosed with a headache secondary to hypertension. Under the physician’s medication and follow-up, his blood pressure reading was maintained at around 140/90 mmHg. Over the subsequent two years, the patient developed episodes of headache daily. At nine years of age, he also underwent tonsillectomy. The patient’s family history revealed that his father had hypertension, frequent headaches secondary to hypertension, and coronary heart disease. Despite several consultations with multiple physicians, a definitive diagnosis was not reached. 

Clinical examination

**Figure 1:**
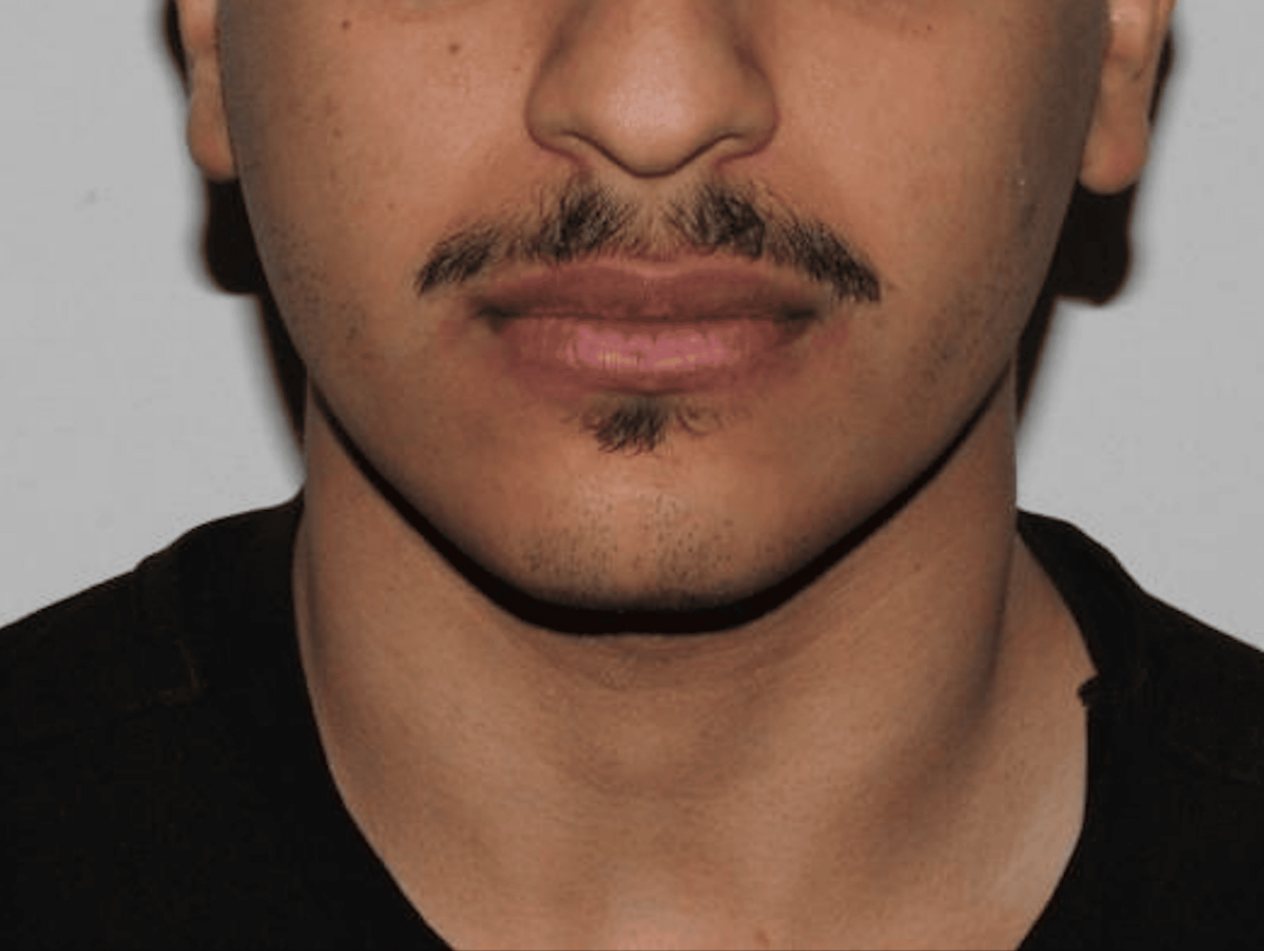
Extraoral image of the patient showing no visible deformity

A routine examination was performed in the dental clinic. On extraoral examination, no visible deformity was evident (Figure 1).

However, tenderness was evident on gentle palpation of the tonsillar fossa. Provocation maneuvers revealed that tilting the head backward or turning it to the left elicited a sharp, stabbing pain in the posterior neck. By contrast, tilting the head forward was identified as the most comfortable position for the patient. To rule out deficits, a comprehensive cranial nerve examination was performed. The pain was traced along the glossopharyngeal nerve distribution.

Diagnosis

**Figure 2:**
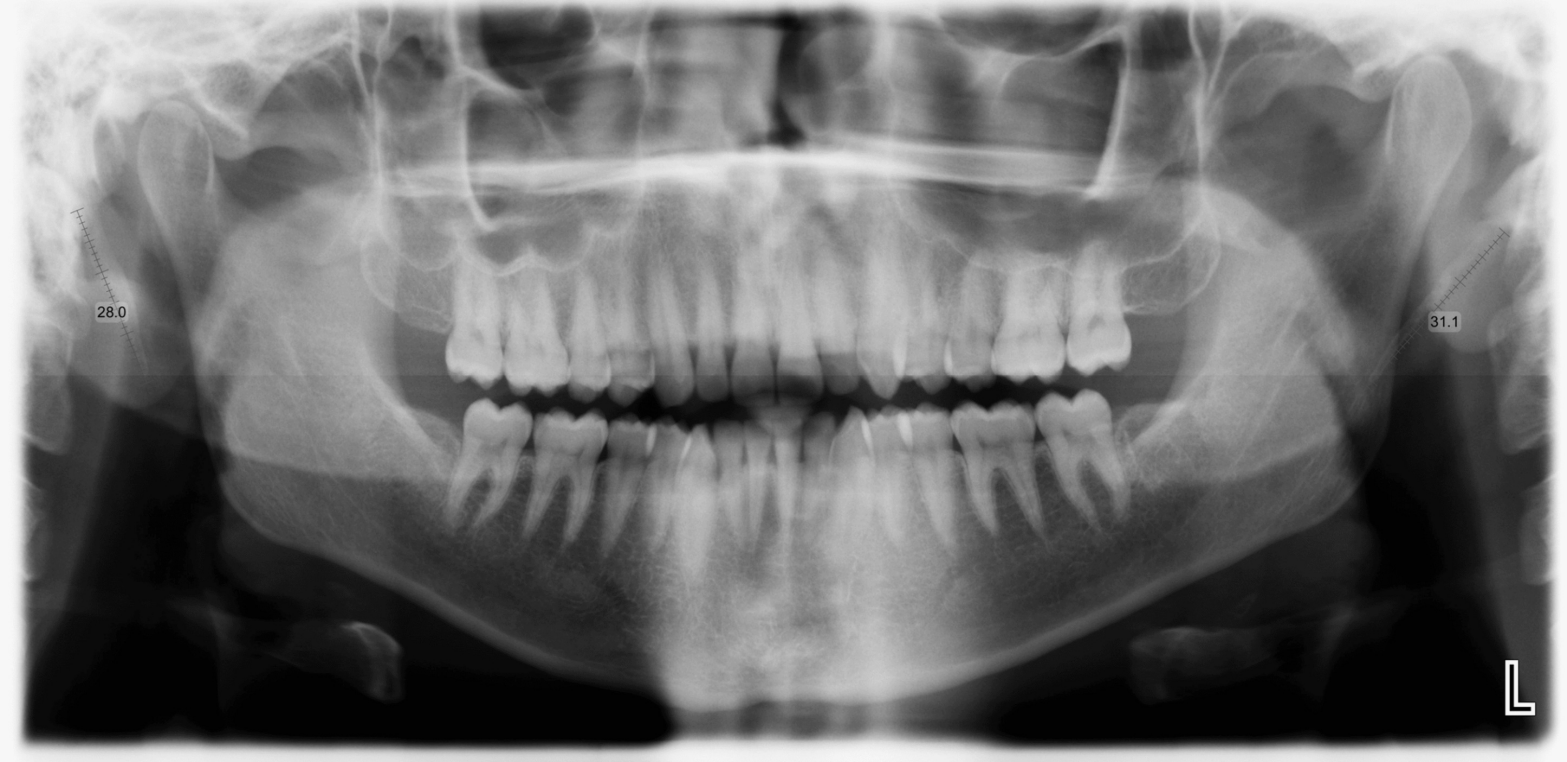
Orthopantamograph showing a bilateral elongated styloid process

An orthopantamograph showed a definitive bilateral elongated styloid process (Figure 2).

**Figure 3:**
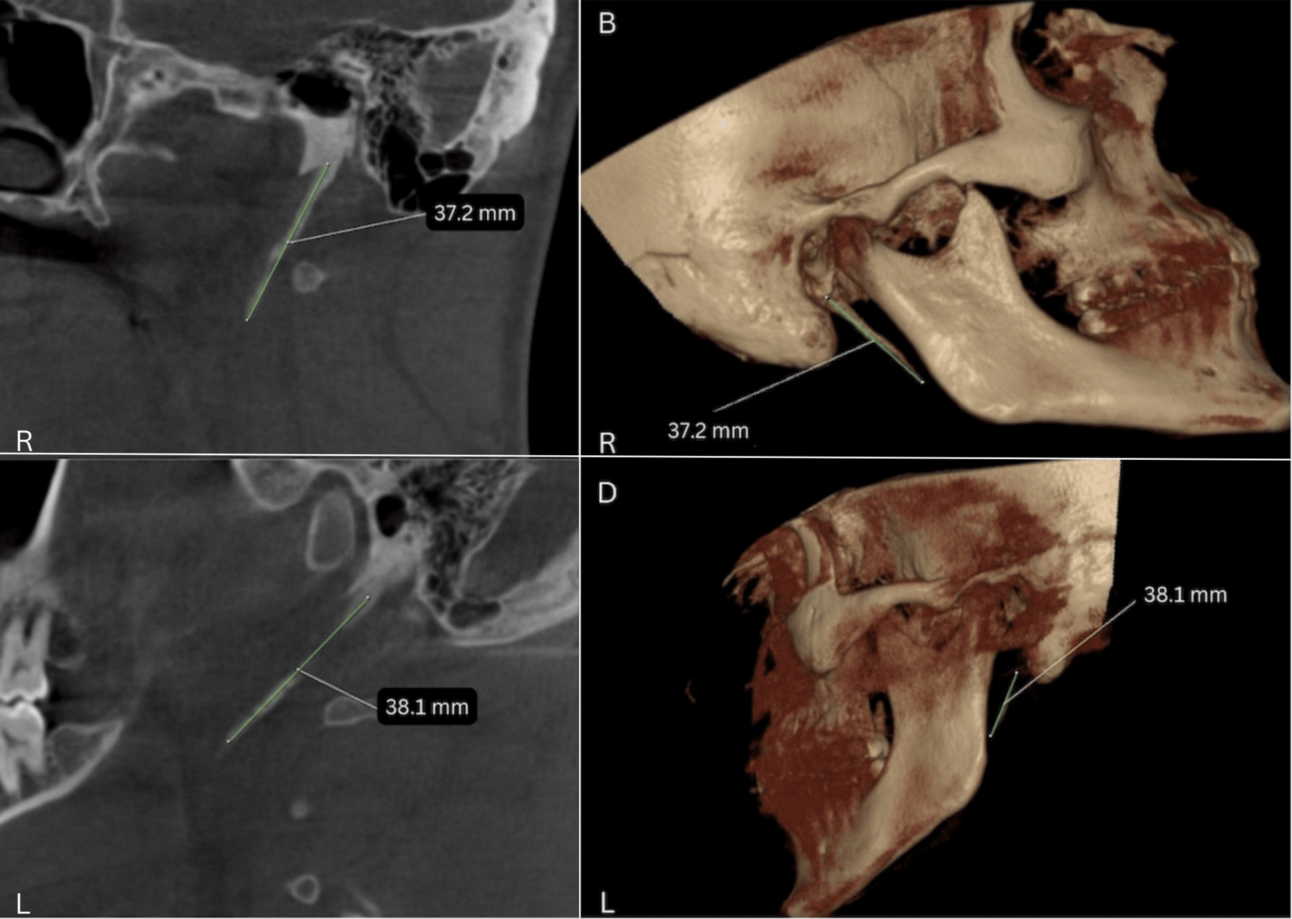
Figures 3A and 3B show the right-side coronal section and a 3D reconstructed image with a styloid process measuring 37.2 mm. Figures 3C and 3D show the left-sagittal section and a 3D reconstructed image with a styloid process measuring 38.1 mm.

Furthermore, a cone-beam computed tomography (CBCT) was done. Figures 3A-3B show the right-side styloid process measuring 37.2 mm. Figures 3C-3D show the left-side styloid process measuring 38.1 mm.

A few differentials were made based on the history, clinical examination, and imaging results. Glossopharyngeal neuralgia is considered as it presents with sharp, radiating pain in the throat and ear. Temporomandibular joint (TMJ) disorder is another differential that can cause ear pain, jaw tenderness, and headache. However, the imaging results did not suggest any of these disorders. Moreover, trigeminal neuralgia was excluded because of the absence of a trigger zone, triggering events, and electric shock-like pain along the trigeminal nerve. Finally, cervical spine disorders such as cervical spondylosis can cause referred pain in the head and neck. However, the absence of characteristic findings for these conditions and the distinct tenderness over the styloid process helped narrow the final diagnosis to Eagle’s syndrome.

## Discussion

The styloid process is a thin bony projection from the temporal bone, typically ranging between 2 and 3 cm long [5]. The syndrome is more prevalent in females between 30 and 50 years [6]. This prevalence makes our case unique as it is a 24-year-old male patient. Embryologically, it arises from the second pharyngeal arch, which is called the Reichert's cartilage. Eagle syndrome is an abnormally elongated styloid process or an ossified styloid ligament with or without an aberrant direction. An elongated styloid process develops from variation in embryological development. However, a calcified stylohyoid ligament occurs as a result of tonsillectomy [7]. An acute angle of the styloid process creates an aberrant direction as it lies close to the pharyngeal wall, inducing pain in this syndrome [8]. The pain may also be secondary to compression or irritation of the adjacent vessels or muscles [5]. Non-specific symptoms, unclear etiology, and failure to diagnose this condition quickly can deteriorate the patient’s quality of life and push them to multiple physicians. Variations in embryological development may cause an elongated styloid process. However, a calcified stylohyoid ligament is expected to result from post-tonsillectomy or traumatic scarring. We suspect this is the case, as the patient had a history of tonsillectomy [7]. There are several theories to the pathogenesis of this condition [9]. Following tonsillectomy, scar tissue may form around the styloid process, causing pain. Alternatively, an elongated styloid process may compress the cranial nerves, such as the glossopharyngeal nerve, resulting in pain in the throat and neck; another possibility is that the styloid process could compress the internal carotid artery, leading to transient ischemic attacks or an effect the sympathetic nerves alongside the artery. 

Eagle's syndrome is marked by symptoms like oropharyngeal pain, difficulty swallowing, and referred pain in the ear and throat. The underlying scientific explanation for the symptoms related to Eagle's syndrome is based on the anatomical connections and the irritation or compression of adjacent structures, such as cranial nerves and the internal carotid artery. The literature also indicates that variations in the styloid process length, often exceeding 30 mm, can result in mechanical irritation or compression of adjacent soft tissues, potentially provoking pain responses [5]. Furthermore, inflammatory alterations in the nearby tissues might worsen the symptoms [10]. Langlais classified the elongated styloid process into three types: type I, which is elongated; type II, which is pseudo-articulated; and type III, which is segmented. The pattern of calcification is also classified into three types: calcified outline, partially calcified, nodular, and completely calcified [11]. In our case, the right side is classified as type I with a completely calcified subtype, and the left is classified as type III with a calcified outline.

Table 1 shows a comparison chart to identify the key findings of our patient that were unique from a classical description of Eagle's syndrome. 

**Table 1:** Comparison of typical symptoms of classical Eagle's syndrome and the case presented.

Typical symptoms of classical Eagle's syndrome	Presented case
Common in females	The case presented was a male.
Common between 30 and 50 years	The case presented was 24 years old.
Symptoms include oropharyngeal pain, difficulty swallowing, referred pain in the ear and throat, pain on turning the head, and foreign body sensation in the throat.	The case presented had a headache localized to the left side of the forehead, pain when turning the head, and an electric shock-like sensation on the back of the neck.
Positive history of tonsillectomy	The patient had a tonsillectomy done nine years back.
Tenderness on palpation of tonsillar fossa	Tenderness on palpation of tonsillar fossa of the patient.
The elongated styloid process confirmed in imaging studies	A panoramic radiograph and cone beam computed tomography confirm the elongated styloid process.

The treatment options can be nonsurgical or surgical, depending on the severity of symptoms and the patient’s response to initial treatments. Nonsurgical approaches include medications such as analgesics, nonsteroidal anti-inflammatory drugs (NSAIDs), antiepileptics, or muscle relaxants. Injections of local anesthetics or steroids into the styloid process may be used to alleviate pain [10]. While these treatments are more conservative, they are unfortunately associated with a higher likelihood of symptom recurrence, typically within six to 12 months following treatment [12].

Surgical treatment is reserved for patients with significant pain and is unresponsive to conservative measures. Styloidectomy is a surgical procedure to remove or shorten the styloid process. Styloidectomy can be performed through either an intraoral or an extraoral approach. An intraoral approach is less invasive but carries a higher risk of infection and potential nerve damage. The external approach, performed through an incision in the neck, provides better visibility and access, reducing the risk of complications. However, it may leave a visible scar [13]. 

Treatment choice depends on the individual’s symptoms and preferences, with surgery reserved for more severe or refractory cases. The most appropriate therapeutic approach for this case is minimally invasive cervical styloidectomy (MICS). Bargiel et al. reported a remarkable success rate of 97% with this procedure [14]. This technique is minimally invasive and highly effective. It is successful in alleviating the associated symptoms. One potential complication that may arise from MICS in bilateral cases of Eagle’s syndrome is temporary facial nerve weakness, which can occur due to retraction during the procedure.

Now that we have arrived at a diagnosis, the patient is currently on symptomatic treatment. Based on his response, surgical intervention may or may not be required. 

## Conclusions

Accurate clinical diagnosis is essential for managing rare conditions like Eagle’s syndrome. A comprehensive patient history, paired with a methodical physical examination, offers crucial insights into the distinctive features of the condition, such as pain elicited by specific head movements or localized sensitivity over the styloid process. Advanced imaging techniques, like CBCT, are valuable to confirm the diagnosis. The takeaway message is that neurologists, otolaryngologists, and dental surgeons should be aware of the incidence of this clinical condition to alleviate their patient’s suffering. The overlapping symptoms may result in misdiagnosis or treatment delays if not thoroughly examined. This case demonstrates that identifying rare conditions requires clinicians to think beyond common pathologies and focus on patient-specific details.
